# The Nutraceutical Potential of Blackcurrant (*Ribes nigrum*) for Glycemic Control and Metabolic Health: Mechanistic and Translational Insights

**DOI:** 10.3390/nu18172852

**Published:** 2026-09-01

**Authors:** Piotr Religa, Magdalena D. Pieczynska-Kovacs, Marzena Łazarczyk, Bhupinder Kapoor, Rajan Logesh, Smith B. Babiaka, Piotr Poznański, Michel-Edwar Mickael, Ricardo Lagoa, Mariusz Sacharczuk, Ibrahim F. Rehan, Asmaa Elnagar, Ana Petra Cherciu, Artur Jóźwik, Atanas G. Atanasov, Jaroslaw Olav Horbańczuk

**Affiliations:** 1 Department of Medicine, Karolinska Institute, Visionsgatan 18, 171 76 Solna, Sweden; 2 Institute of Genetics and Animal Biotechnology, Polish Academy of Sciences, 36a Postepu, 05-552 Jastrzebiec, Poland; 3 School of Pharmaceutical Sciences, Lovely Professional University, Phagwara 144411, Punjab, India; 4 Department of Pharmacognosy, Faculty of Pharmacy, M.S. Ramaiah University of Applied Sciences, Bengaluru 560054, Karnataka, India; 5 Department of Chemistry, Faculty of Science, University of Buea, Buea P.O. Box 63, Cameroon; 6 Department of Microbial Bioactive Compounds, Interfaculty Institute for Microbiology and Infection Medicine, University of Tübingen, 72074 Tübingen, Germany; 7 Associate Laboratory in Chemical Engineering, School of Technology and Management, Polytechnic Institute of Leiria, 2411-901 Leiria, Portugal; 8 Department of Pathobiochemistry, Faculty of Pharmacy, Meijo University, Tempaku-ku, Nagoya-shi 468-8503, Aichi, Japan; 9 Department of Animal Behavior and Husbandry, Faculty of Veterinary Medicine, Menoufia University, Shebin Alkom 32511, Menoufia, Egypt; 10 St. Luke Chronic Diseases Hospital, Șoseaua Berceni 12, 041915 București, Romania; apcherciu@yahoo.com; 11 Patient Safety & Digital Health (PaDiH) Group, Danube Private University, 3500 Krems-Stein, Austria; 12 Department of Biochemistry, Saveetha Institute of Medical and Technical Sciences, Saveetha Medical College and Hospital, Chennai 600077, Tamil Nadu, India; 13 Ludwig Boltzmann Institute Digital Health and Patient Safety, Medical University of Vienna, 1090 Vienna, Austria

**Keywords:** *Ribes nigrum*, diabetes, gut microbiota, metabolic regulation

## Abstract

Diabetes mellitus is a pervasive global health crisis, with type 2 diabetes (T2D) representing the majority of cases. While conventional pharmacological therapies exist, suboptimal efficacy, adverse effects, and inadequate glycemic control in a substantial proportion of patients highlight the need for complementary strategies. There is growing scientific and clinical interest in plant-based functional foods, including *Ribes nigrum* L. (blackcurrant), which is rich in bioactive phytochemicals such as anthocyanins, flavonols, and phenolic acids. This review evaluates the antidiabetic and metabolic regulatory potential of blackcurrant, focusing on its multi-target mechanisms of action. Available experimental evidence suggests that blackcurrants may influence glucose metabolism through several mechanisms, including inhibition of carbohydrate-digesting enzymes (α-amylase and α-glucosidase), modulation of intestinal glucose transporters (SGLT1 and GLUT2), and potential modulation of insulin sensitivity involving AMPK signaling and GLUT4 translocation. In addition, blackcurrant constituents may stimulate glucagon-like peptide-1 (GLP-1) secretion, modulate gut microbiota composition, and exert antioxidant and anti-inflammatory effects that counteract key drivers of insulin resistance and β-cell dysfunction. Supporting human studies indicate that blackcurrant consumption has been associated with attenuation of postprandial glycemic and insulinemic responses and modest improvements in cardiometabolic biomarkers, although findings remain limited by study size, duration, and formulation heterogeneity. Overall, *Ribes nigrum* represents a promising dietary adjunct for the prevention and management of T2D through multi-target metabolic regulation.

## 1. Introduction

Type two diabetes (T2D) is a serious chronic metabolic disorder. It is the most prevalent type of diabetes mellitus, representing a major global health burden [1,2]. It is characterized by persistent hyperglycemia resulting from impaired insulin secretion, insulin action, or both [3]. Defective insulin signaling disrupts carbohydrate, lipid, and protein metabolism, affecting key tissues, including skeletal muscle, adipose tissue, and liver [4]. Complications of T2D include microvascular complications such as retinopathy, nephropathy, and neuropathy, as well as macrovascular outcomes, including cardiovascular disease and stroke [5,6]. The disease also imposes a substantial mortality burden and an economic burden worldwide.

Many T2D patients require pharmacological therapy to maintain glycemic control [7]. Current treatments include metformin, sulfonylureas, thiazolidinediones, meglitinides, and newer agents such as SGLT2 inhibitors, GLP-1 receptor agonists, and DPP-4 inhibitors [8]. However, its clinical use may be limited by predominantly gastrointestinal adverse effects, particularly at higher doses, while its pharmacological action is primarily restricted to inhibition of intestinal α-glucosidases [9]. These limitations have driven increasing interest in complementary nutritional strategies. Functional foods are perceived to be safe and affordable [10]. Over 1200 plant species have been reported to exhibit antidiabetic effects [11,12,13,14]. Many of these effects could be attributed to the antioxidant, anti-inflammatory, and glucose-lowering properties of polyphenols [15,16].

*Ribes nigrum* (blackcurrant), a polyphenol-rich berry from the Grossulariaceae family, has emerged as a potentially useful functional food for supporting glycemic and metabolic health and T2D therapy [17]. Blackcurrants are native to temperate regions of Europe and Asia and are widely cultivated in Northern Europe and Poland [18,19]. Blackcurrants incorporated into carbohydrate-rich foods have been reported to reduce the glycemic index and postprandial glucose responses in in vitro and human studies, although the magnitude and consistency of these effects vary across formulations and study populations [20,21]. Preclinical evidence suggests that blackcurrants may influence multiple metabolic pathways, including carbohydrate digestion, insulin signaling, gut microbiota, and inflammatory processes [22]. The polyphenol composition of blackcurrants consists of up to 90% anthocyanins, which are responsible for their intense dark purple color and major health properties. The remaining 10% to 40% is made up of a supportive blend of flavonols (such as quercetin), phenolic acids, and tannins [22]. Anthocyanins also inhibit carbohydrate-digesting enzymes and modulate starch structure, contributing to improved postprandial glucose control [23]. Blackcurrant anthocyanins interact with pathways such as AMPK, GLUT4, and incretin signaling, and may complement standard therapies such as metformin [24,25]. In humans, various reports have indicated that anthocyanins can enhance postprandial glycemic responses; the effects seem to vary depending on metabolic status and study design [26]. However, research summarizing blackcurrants’ effects and molecular pathways remains limited.

This review aims to address this challenge by providing a comprehensive, multi-target discussion of the current literature on *Ribes nigrum*. By linking its specific phytochemical composition to organ-specific metabolic pathways, including carbohydrate digestion, intestinal transport, peripheral glucose uptake, incretin kinetics, inflammation, and gut microbiota regulation, we aim to evaluate its clinical efficacy and the translational challenges it poses. Ultimately, this work seeks to clarify the role of blackcurrants as an evidence-based, complementary dietary strategy in the global prevention and management of T2D.

## 2. Methods

This narrative review was conducted through a structured literature search of PubMed/MEDLINE, Scopus, and Web of Science. The final search was performed on 29 May 2026. The search strategy combined the terms ”*Ribes nigrum*” OR “blackcurrant” with “diabetes”, “glycemic”, “insulin” or “gut microbiota”, “bioavailability”, “pharmacokinetics”, “anthocyanin” and “metabolic syndrome” using appropriate Boolean operators. Reference lists of retrieved articles were screened manually to identify additional publications. The retrieved literature was screened for relevance based on title, abstract, and, where appropriate, full-text assessment. Studies were eligible when they investigated *Ribes nigrum* or blackcurrant-derived preparations and reported relevant metabolic, glycemic, pharmacokinetic, bioavailability, microbiome, or mechanistic outcomes. Studies reporting no relevant metabolic or mechanistic outcome were excluded. Priority was given to primary experimental studies (in vitro, animal, and human) directly investigating *Ribes nigrum* or its derivatives; where evidence was derived from mixed-berry formulations or from anthocyanin sources other than blackcurrants, this was explicitly identified in the text to distinguish blackcurrant-specific findings from evidence extrapolated from other sources. Evidence was categorized as direct when the intervention or experimental material was derived from *Ribes nigrum*, and as indirect when findings were obtained using isolated compounds, other anthocyanin-rich fruits, or related plant-derived preparations. Evidence quality was assessed according to study design and methodological characteristics. For human intervention studies, particular consideration was given to randomization, control/placebo groups, sample sizes, intervention durations, participant characteristics, outcome measures, and potential sources of bias. For human studies, the metabolic status of participants and duration of intervention were specifically considered when interpreting the strength and translational relevance of the evidence. Acute postprandial responses observed in healthy participants were distinguished from longer-term clinical outcomes in individuals with type 2 diabetes, and findings from acute or short-term studies were not interpreted as evidence of sustained improvements in glycemic control or metabolic health. For animal and in vitro studies, study designs, experimental controls, model characteristics, doses/exposures, and reproducibility of reported outcomes were considered. Because this article is a narrative review rather than a systematic review or meta-analysis, no formal standardized risk-of-bias assessment was performed. The strength of evidence was therefore interpreted in relation to the study design, consistency of findings, methodological characteristics, and translational relevance.

## 3. Phytochemical Composition of *Ribes nigrum*

**Figure 1:**
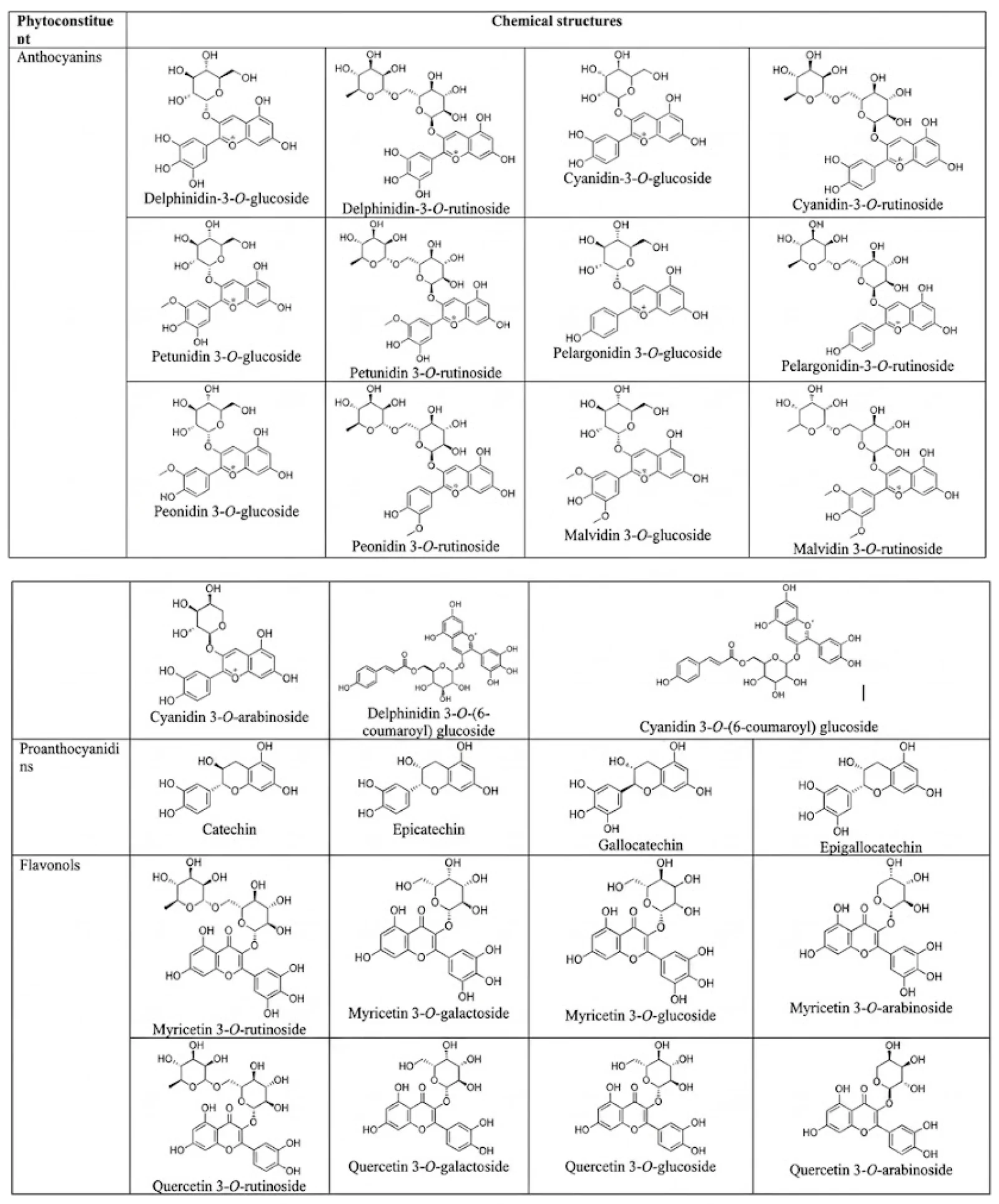
Polyphenolic composition of *Ribes nigrum*. Major polyphenolic compounds identified in blackcurrants comprise anthocyanins (delphinidin, cyanidin, petunidin, pelargonidin, peonidin, and malvidin glycosides), proanthocyanidins (procyanidins and prodelphinidins), and flavonols (myricetin and quercetin glycosides).

*Ribes nigrum* is rich in bioactive phenolic compounds, including anthocyanins, flavonols, flavan-3-ols, and phenolic acids [17]. Among these, anthocyanins, proanthocyanidins, and flavonols represent the major polyphenolic classes and are collectively referred to as blackcurrant flavonoids [18,27,28,29]. (i) The principal anthocyanins identified in *R. nigrum* include delphinidin-3-O-glucoside, delphinidin-3-O-rutinoside, cyanidin-3-O-glucoside, and cyanidin-3-O-rutinoside, along with other derivatives such as petunidin-, pelargonidin-, peonidin-, and malvidin-based glycosides, as well as acylated forms, including delphinidin 3-O-(6-coumaroyl) glucoside and cyanidin 3-O-(6-coumaroyl) glucoside [18] (Figure 1). (ii) In addition to anthocyanins, blackcurrants contain significant amounts of proanthocyanidins, primarily procyanidins and prodelphinidins [30]. Procyanidins are oligomeric and polymeric structures composed of (+)-catechin and (−)-epicatechin units, whereas prodelphinidins are derived from (+)-gallocatechin and (−)-epigallocatechin subunits. Flavonols present in blackcurrants are mainly represented by myricetin, quercetin, and kaempferol derivatives [31,32]. In addition to polyphenols, blackcurrant berries contain organic acids, including malic, citric, quinic, and ascorbic acid, as well as sugars such as fructose, glucose, and sucrose [33]. Fresh *R. nigrum* fruit is also a rich source of essential vitamins, including vitamins C, K, B-complex, A, and E, with vitamin C being the most abundant, typically ranging from 125 to 151 mg/100 g fresh weight [34,35].

## 4. Mechanisms of Action Relevant to Glycemic and Metabolic Regulation

Blackcurrants have been proposed to influence glucose and metabolic homeostasis through multiple complementary pathways, although the strength of evidence varies considerably among mechanisms. Preclinical studies suggest potential effects on (i) carbohydrate-digesting enzymes, including α-glucosidase and α-amylase; (ii) intestinal glucose transporters, including SGLT1 and GLUT2; (iii) peripheral glucose uptake through the AMPK–GLUT4 axis in skeletal muscle; (iv) GLP-1 secretion and glucose tolerance; (v) gut microbiota composition and short-chain fatty acid (SCFA)-related signalling; and (vi) oxidative stress and NF-κB-mediated inflammation. Human studies provide clinical support primarily for attenuation of postprandial glycemic and insulinemic responses, whereas direct evidence for the specific molecular mechanisms remains limited. Accordingly, the mechanisms discussed below are interpreted according to the available level of evidence, with findings from in vitro and animal studies distinguished from those demonstrated in humans [36].

### 4.1. Modulation of Carbohydrate Digestion Enzyme Pathways

**Figure 2:**
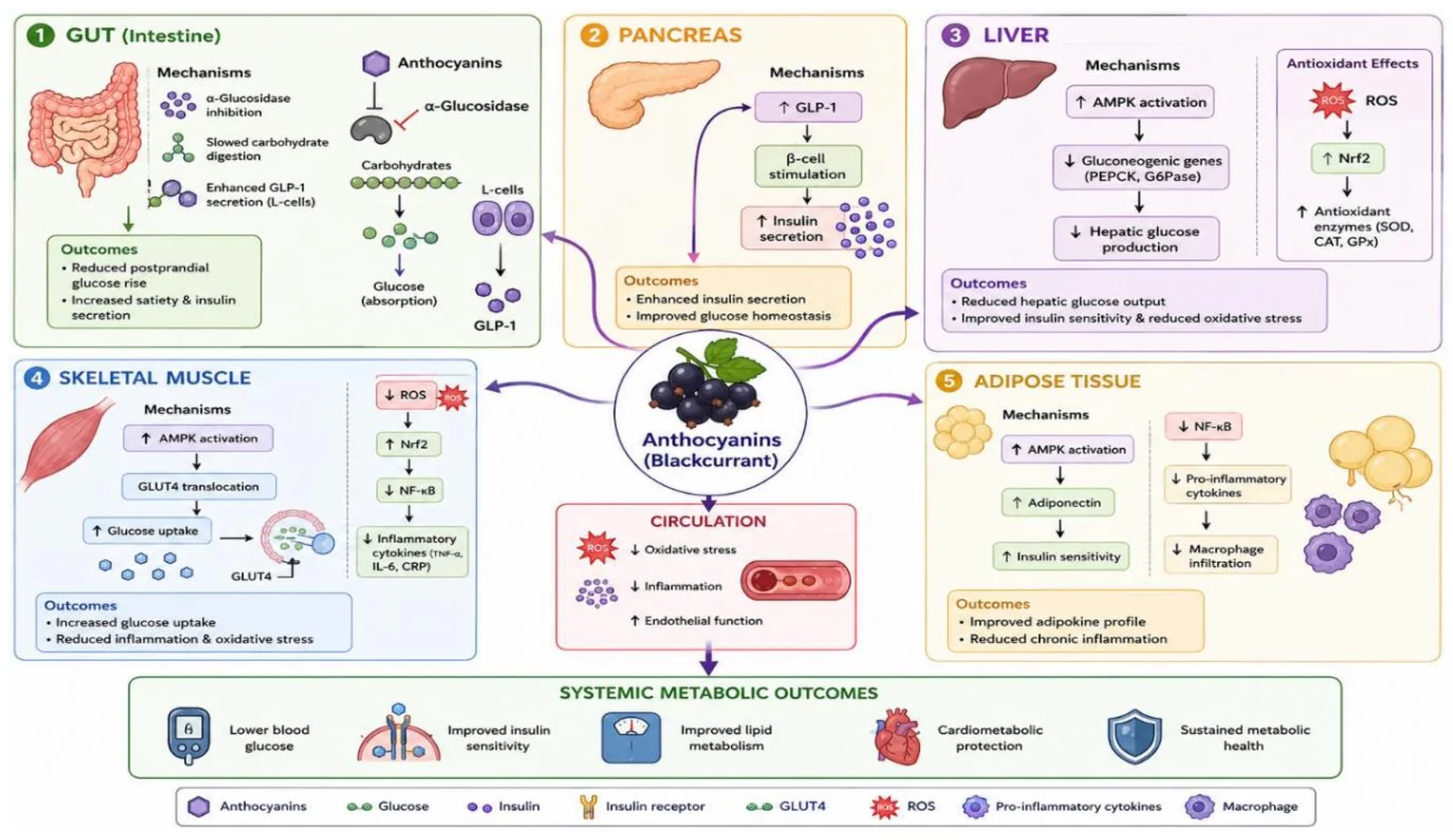
Organ-specific metabolic actions of blackcurrant-derived anthocyanins across the gut, pancreas, liver, skeletal muscle, and adipose tissue, highlighting AMPK activation, GLUT4 translocation, GLP-1 secretion, α-glucosidase inhibition, and modulation of inflammatory and oxidative-stress signaling, which together support improved glucose uptake, reduced hepatic glucose production, and enhanced insulin sensitivity.

Blackcurrants may influence postprandial glycemia by modulating the activity of several carbohydrate-digesting enzymes that play a major role in carbohydrate digestion [29,37]. These enzymes include (i) α-amylase, which is an enzyme that is important for initiating starch hydrolysis in the oral cavity (Figure 2 and Table 1) [38,39] and (ii) α-glucosidase, which is an intestinal enzyme that breaks down complex carbohydrates into glucose [40]. Its activity directly influences postprandial blood sugar levels; inhibiting this enzyme slows carbohydrate digestion in the small intestine, thus reducing the rate of glucose absorption into the bloodstream and blunting sharp postprandial glucose excursions [9]. It has been shown that blackcurrant phenolic acids and flavonols contribute to the modulation of salivary amylase [41]. Xiaodan Hui et al. showed that blackcurrant powder significantly enhances the inhibition of α-amylase when added to oat bran. This effect was reflected by reduced IC_50_ values, indicating stronger inhibitory activity and therefore greater potential to slow starch and sugar breakdown. Notably, blackcurrant extract acted as a competitive inhibitor of α-amylase, suggesting a direct interaction with the enzyme’s active site. These effects were linked to its high content of anthocyanins, especially cyanidin and delphinidin derivatives, which showed strong binding affinity through hydrogen bonding interactions in molecular docking analyses [41]. Competitive inhibition may explain the reason why McDougall et al’s blackcurrant polyphenols exert moderate rather than complete inhibition of α-amylase activity, thereby slowing starch hydrolysis and glucose release without fully impairing carbohydrate digestion [27,42]. This regulatory effect contrasts with the action of pharmacological inhibitors such as acarbose, where potent α-amylase suppression is linked to gastrointestinal discomfort due to undigested starch fermentation in the colon. (ii) In the case of α-glucosidase, Boath et al. demonstrated that polyphenol-rich berry extracts can inhibit α-glucosidase activity in vitro, with blackcurrant extract showing particularly strong effects. The blackcurrant extract, dominated by anthocyanins (~70% of total polyphenols), inhibited α-glucosidase with an IC_50_ of about 20 μg GAE/mL, performing as effectively as the pharmaceutical inhibitor acarbose. Importantly, it enhanced the inhibitory action of acarbose [43]. Furthermore, studies of other anthocyanin-rich fruits have reported α-glucosidase inhibitory activity comparable to that of acarbose. For example, Takikawa et al. [44] investigated bilberries rather than blackcurrants and therefore provide indirect evidence regarding the potential inhibitory effects of anthocyanin-rich fruits on carbohydrate-digesting enzymes [44]. The inhibitory activity appears to be dose-dependent and often synergistic, as whole-fruit extracts outperform isolated anthocyanins [43,45].

### 4.2. Modulation of SGLT1 and GLUT2 Pathways

Blackcurrant-associated polyphenols may modulate glucose uptake via the enterocyte glucose transporters SGLT1 and GLUT2 in various organs. SGLT1 and GLUT2 are key glucose transporters involved in absorption and regulation of blood sugar [54]. In the intestine, SGLT1 actively absorbs glucose and galactose using sodium gradients, while GLUT2 helps move glucose into the blood, regulates glucose sensing in the pancreas for insulin release, and balances glucose storage and release in the liver [55,56]. In diabetes, both transporters become dysregulated: SGLT1 and GLUT2 are overexpressed in the gut, causing excessive glucose absorption; GLUT2 mislocalizes to enhance sugar uptake; pancreatic glucose sensing is impaired, leading to poor insulin secretion; and renal SGLT1 increases glucose reabsorption, all of which worsen hyperglycaemia [57,58]. Kwon et al. investigated whether intestinal glucose absorption mediated by the transporter GLUT2 could be inhibited by flavonoids. Using heterologous expression systems (such as *Xenopus laevis* oocytes) expressing GLUT2, they found that these flavonoids produced strong inhibition of both glucose and fructose transport. The study examined isolated flavonoids rather than blackcurrant or a *Ribes nigrum*-derived preparation; these findings should be considered indirect mechanistic evidence and cannot be interpreted as direct evidence of GLUT2 inhibition by blackcurrants. Furthermore, the inhibition was noncompetitive, indicating that flavonoids do not block the sugar-binding site directly but likely interfere with transporter function through an allosteric or membrane-related mechanism. The potency was high, with IC_50_ values for quercetin, myricetin, and isoquercitrin being several hundred- to a thousand-fold lower than physiological sugar concentrations. These findings demonstrate the potential of flavonoids to modulate GLUT2 activity in vitro; however, their physiological relevance to blackcurrant consumption remains to be established [42,46]. Moreover, dual SGLT1/SGLT2 inhibitors such as sotagliflozin are known to block both renal reabsorption through SGLT2 and intestinal absorption through SGLT1 [59,60,61]. However, sotagliflozin carries a higher risk of gastrointestinal side effects, specifically diarrhea, alongside serious metabolic and infectious risks [62]. Whether blackcurrant-derived flavonoids can provide clinically relevant modulation of intestinal glucose transport at achievable dietary concentrations, or interact beneficially with established glucose-lowering therapies remains unknown and warrants further investigation in controlled experimental and clinical studies.

### 4.3. Restoration of GLUT4-Mediated Glucose Uptake Through AMPK

Preclinical evidence suggests that blackcurrants may influence peripheral glucose disposal, potentially through enhanced GLUT4 (glucose transporter type 4) translocation. Available experimental data further suggest that AMPK (AMP-activated protein kinase) signaling may contribute to this effect, although the relative contribution of AMPK-dependent and canonical insulin-signaling pathways remains to be established. GLUT4 is an insulin-responsive glucose transporter mainly in skeletal muscle and adipose tissue that, in basal conditions, is stored in intracellular vesicles and must translocate to the plasma membrane to enable glucose uptake, making muscle GLUT4 trafficking a key determinant of postprandial glucose clearance [63,64,65]. This translocation is regulated by two main pathways: insulin signaling, which activates the IR–IRS-1–PI3K–Akt cascade, leading to Akt-mediated inhibition of AS160 (TBC1D4) and the release of Rab-dependent vesicle trafficking, and an insulin-independent pathway that is mediated by AMPK [66]. AMPK is activated by an increased AMP:ATP ratio via LKB1 or CaMKK2, which promotes GLUT4 movement through AS160 and TBC1D1 [67]. In type 2 diabetes, insulin signaling is impaired and is associated with decreased GLUT4 translocation combined with a reduction in its expression in the adipose tissue [63]. In contrast, AMPK signaling is relatively preserved and can still drive GLUT4 translocation, providing an alternative route for glucose uptake when insulin signaling is defective [68,69]. Blackcurrants appear to act on this particular pathway. In type 2 diabetic KK-Ay mice, dietary blackcurrant extract demonstrated a significant increase in phospho-AMPKα (threonine-172) in skeletal muscle, together with increased GLUT4 at the muscle plasma membrane, and these changes were accompanied by reduced blood glucose and improved glucose tolerance [47]. This route is engaged by the principal antidiabetic agents. Metformin is reported to lower glycaemia in substantial part through LKB1-dependent activation of AMPK, which may promote GLUT4-dependent uptake in muscle while suppressing hepatic gluconeogenesis [70].

### 4.4. Modulation of the GLP-1 Insulin Axis

Preclinical and experimental evidence suggests that blackcurrants can enhance the incretin axis by stimulating GLP-1 secretion, an effect thought to be driven mainly by its major anthocyanin, delphinidin-3-rutinoside (D3R). GLP-1 is a gut-derived incretin hormone produced by enteroendocrine L-cells of the distal small intestine and colon that augments meal-stimulated insulin secretion and is rapidly degraded by DPP-4 [71]. Under normal conditions, GLP-1 release is triggered either by direct nutrient sensing, including glucose uptake through SGLT1 and fatty-acid sensing via GPR40 and GPR120, or by sensing microbial metabolites, particularly SCFAs produced by colonic fermentation that activate FFAR2 and FFAR3 [72]. Secreted GLP-1 enhances glucose-dependent insulin secretion, suppresses glucagon release, slows gastric emptying, and promotes satiety, collectively limiting postprandial glucose excursions [73]. In type 2 diabetes, the incretin effect is reduced, with diminished insulin secretion after oral versus intravenous glucose, potentially due to reduced GLP-1 secretion, impaired responsiveness, or both, contributing to inadequate postprandial insulin release and weaker satiety signalling [74]. Blackcurrants appear to act on the secretory side of this axis through D3R, which may function as a direct GLP-1 secretagogue. In GLUTag cells (a murine enteroendocrine L-cell line), D3R was shown to stimulate GLP-1 secretion through the Ca2+/CaMKII (calcium/calmodulin-dependent protein kinase II) pathway, possibly involving GPR40/120 [48]. In rats, a pre-administered D3R-rich blackcurrant extract (BCE) ameliorated glucose tolerance by stimulating GLP-1 secretion and subsequent insulin release, and D3R was found to remain largely intact in the gastrointestinal tract for at least 45 to 60 min, which suggests that the effect may be mediated by the intact anthocyanin rather than by its degradation products [49]. In type 2 diabetic KK-Ay mice, dietary BCE increased basal plasma GLP-1 with concurrent upregulation of PC1/3 (prohormone convertase 1/3), the enzyme that processes intestinal proglucagon to GLP-1. These mechanistic parallels map onto established drug actions in kind but not in degree: α-glucosidase inhibition parallels acarbose, with likely better gastrointestinal tolerance; AMPK activation parallels metformin; and GLP-1 stimulation parallels incretin therapies, yet in every case the effect is far smaller and largely preclinical [75].

### 4.5. Regulation of Oxidative Stress and NF-κB Inflammatory Signaling

Blackcurrant delphinidin- and cyanidin-based glycosides scavenge superoxide, hydroxyl radicals, and peroxynitrite [18]. Additional mechanistic evidence from other anthocyanin-rich sources supports the antioxidant potential of these compounds. For example, Noda et al. [76] reported that pomegranate-derived anthocyanins and isolated anthocyanidins exhibited radical-scavenging activity; however, because this study investigated pomegranate and isolated compounds rather than *Ribes nigrum*, these findings represent indirect evidence and should not be interpreted as direct evidence for the antioxidant effects of blackcurrants. Chronic oxidative stress and low-grade inflammation are central to insulin resistance and β-cell dysfunction [77]. Excess nutrient flux promotes reactive oxygen species (ROS) generation, activating the nuclear factor kappa B (NF-κB) pathway and raising pro-inflammatory cytokines, including TNF-α, IL-6, and MCP-1 [78]. In an interesting study, Benn et al. investigated whether blackcurrant extract could prevent obesity-associated inflammation using male C57BL/6J mice fed a high-fat/high-cholesterol diet for 12 weeks, either alone or supplemented with 0.1% blackcurrant extract. Blackcurrant extract supplementation significantly reduced epididymal fat mass, adipocyte size, and the number of crown-like structures (CLS), a histological marker of macrophage infiltration and adipose tissue inflammation. These changes were accompanied by lower expression of inflammatory markers, including F4/80, CD68, and IKKε, with F4/80 and IKKε levels positively correlating with CLS abundance. In skeletal muscle, blackcurrant extract increased the expression of genes linked to energy expenditure and mitochondrial biogenesis, including PPARα, PPARδ, UCP-2, UCP-3, and mitochondrial transcription factor A. Furthermore, lipopolysaccharide-stimulated splenocytes from blackcurrant extract-fed mice exhibited reduced expression of the pro-inflammatory cytokines TNF-α and IL-1β [50,51]. These findings indicate that blackcurrant extract might be beneficial in attenuating chronic oxidative stress and low-grade inflammation in diabetes.

### 4.6. Regulation of the Microbiota SCFA Pathway

The interaction between blackcurrant, the gut microbiota, and microbiota-derived short-chain fatty acids (SCFAs) may contribute to its metabolic effects in T2D. Colonic microbiota ferments the carbohydrate that escapes enzymatic digestion and transporter-mediated absorption in the upper gut into short-chain fatty acids (SCFAs), predominantly acetate, propionate, and butyrate. Because blackcurrants may slow upper-gut carbohydrate digestion and absorption, it has been hypothesized that it could increase the amount of carbohydrate reaching the colon for microbial fermentation [79]. In turn, SCFAs act on the L-cell receptors FFAR2 and FFAR3 to promote GLP-1 secretion and maintain barrier integrity by limiting the translocation of bacterial products and the low-grade metabolic endotoxemia that drives NF-κB-mediated inflammation and insulin resistance [80]. The microbiota may also underlie part of the systemic action attributed to blackcurrant extract in peripheral tissues, since much of the bioavailable anthocyanin exposure consists of microbiota-derived phenolic metabolites rather than the poorly absorbed parent compounds [81,82,83]. Type 2 diabetes and obesity are accompanied by dysbiosis, with reduced diversity, a loss of SCFA-producing taxa, and impaired barrier function, and the first-line agent metformin is itself a microbiota-modifying drug, providing a pharmacological precedent for this target [84,85]. Blackcurrant extract has been reported to alter microbial community composition across several models, although the taxa differ between studies and species: in mice, long-term supplementation modified gut microbiome profiles in an age-dependent manner; in non-obese type 2 diabetic rats, an aqueous extract modulated the microbiome in association with improved insulin sensitivity and secretion; and in the single available human study, a blackcurrant product increased Lactobacilli and Bifidobacteria and decreased β-glucuronidase activity and faecal pH [52,53]. These connections position the microbiota as a plausible hub linking the gut, incretin, inflammatory, and peripheral arms.

## 5. Clinical Evidence in Humans

Human studies provide the strongest translational evidence for the metabolic effects of blackcurrants, although the available clinical evidence remains limited. Randomized and controlled studies indicate that blackcurrant consumption can attenuate postprandial glycemic and insulinemic responses, while repeated intake may produce modest improvements in insulin sensitivity, oxidative-status markers, and selected gut microbiota parameters. However, most studies are small and short in duration, and several frequently cited trials use mixed-berry formulations, limiting attribution of the observed effects specifically to blackcurrants. Importantly, the human evidence primarily supports metabolic outcomes rather than the specific molecular mechanisms proposed in preclinical studies.

### 5.1. Acute Postprandial Studies

An acute, single-dose intake of blackcurrants consistently lowers early postprandial glucose and insulin in healthy adults. A randomized, double-blind crossover trial of drinks providing 150, 300, or 600 mg blackcurrant anthocyanins before a high-carbohydrate meal reduced early postprandial glucose and insulin at the highest dose, with parallel lowering of incretin (GIP and GLP-1) secretion. A related trial showed the same effect for blackcurrant polyphenol drinks [86,87] (Table 2). A 75 g blackcurrant purée, alone or with fermented quinoa, similarly reduced glucose and insulin during the first 30 to 60 min, and sucrose co-ingested with blackcurrants and lingonberries prevented late postprandial hypoglycemia in healthy women [20,21]. These observations are consistent with the delayed glucose appearance expected from α-amylase and α-glucosidase inhibition and from reduced SGLT1-mediated uptake [88]. However, a single 600 mg dose of New Zealand blackcurrant extract produced no acute change in postprandial glucose, insulin, or triglycerides, and a blackcurrant and citrus drink produced only modest acute effects, indicating that the acute response is real but dose- and formulation-dependent rather than universal [26].

### 5.2. Longer-Term and Metabolic-Risk Studies

Although improvements in insulin sensitivity are documented in vitro, in animal models, and in some short-term human studies, a repeated intake of blackcurrant extract produces only modest gains in insulin sensitivity, oxidative status, and microbiota composition in people. The clearest blackcurrant-specific result is that eight-day supplementation with the New Zealand blackcurrant extract improved insulin sensitivity by 22% and lowered C-reactive protein [89]. Five-week consumption of an anthocyanin-rich blackcurrant extract reduced oxidative-stress markers and raised plasma antioxidant capacity [90]. A seven-day blackcurrant beverage increased plasma antioxidant capacity in healthy adults [93]. A four-week blackcurrant intervention increased Lactobacilli and Bifidobacteria and lowered β-glucuronidase activity and stool pH, consistent with microbiota modulation in humans on surrogate endpoints [52]. Twelve-week trials reporting changes in adipokines, HbA1c, and lipid parameters in prediabetic or newly diagnosed diabetic cohorts used purified bilberry and blackcurrant anthocyanins together, so their benefits cannot be attributed to blackcurrants specifically [91,92]. Interpreted as a whole, these mechanisms map onto established drug actions in kind but not in degree: α-glucosidase inhibition parallels acarbose, with likely better gastrointestinal tolerance; AMPK activation parallels metformin; and GLP-1 stimulation parallels incretin therapies, yet in every case, the effect is far smaller and largely preclinical [75].

These modest clinical effects are best understood in light of blackcurrant pharmacokinetics, which impose a ceiling on potency. Parent blackcurrant anthocyanins are poorly bioavailable: even a large 2380 mg anthocyanin dose produced a peak plasma concentration of only about 147 nM at 1.5 h, and urinary recovery of intact anthocyanins is typically 0.04 to 0.11% of the ingested dose [94]. Their metabolites circulate at far higher levels: stable-isotope tracing places the true relative bioavailability of cyanidin-3-glucoside near 12%, well above the under-1% implied by parent-compound recovery, and phenolic metabolites such as protocatechuic, ferulic, vanillic, and hippuric acids reach plasma concentrations of up to roughly 2000 nM and persist for many hours, far longer and higher than the parent anthocyanins. Because these metabolites are partly microbiota-derived, inter-individual differences in the gut microbiome are a plausible source of the variable clinical responses observed across the human trials [80].

Taken together, the clinical evidence should be interpreted cautiously. Most human studies are limited by small sample sizes, short intervention periods, and substantial heterogeneity in blackcurrant formulations, anthocyanin doses, food matrices, intervention durations, and participant characteristics. Moreover, several studies use mixed-berry or multi-component formulations, making it difficult to attribute observed effects specifically to blackcurrants. The available trials also differ considerably in metabolic status, ranging from healthy individuals to overweight, prediabetic, or diabetic participants, which may contribute to differences in treatment response. Furthermore, many studies assess acute postprandial responses or surrogate biomarkers rather than clinically meaningful long-term outcomes such as sustained changes in HbA1c or diabetes progression. Consequently, current human evidence supports the potential of blackcurrants as a dietary adjunct but remains insufficient to establish consistent clinical efficacy or to define an optimal formulation, dose, or duration of use for glycemic management.

## 6. Limitations and Future Directions

Several constraints must be resolved before clinical translation. First, blackcurrant phytochemical profiles vary markedly with genotype, cultivation, climate, ripening, storage, and processing, and differences in extraction and food matrix hinder standardization; therefore, the biological effects differ across juices, powders, fermented products, and purified extracts [33,35]. Importantly, the total anthocyanin content alone may not adequately capture biological activity, as the relative proportions of individual anthocyanins, their glycosylation patterns, accompanying polyphenols, and subsequent metabolite formation may influence bioavailability and biological effects. This variability complicates the translation of phytochemical composition into reproducible clinical exposure and metabolic responses. Second, and most fundamentally, the low and variable bioavailability of parent anthocyanins, the dominant role of microbiota-derived phenolic metabolites, and the resulting dependence of response on individual microbiome composition mean that the relationship between an ingested dose and a clinically meaningful tissue effect is still poorly defined [95]. Third and most importantly, the clinical evidence remains insufficient to support strong therapeutic recommendations. Human studies are generally characterized by small sample sizes, short intervention periods, heterogeneous blackcurrant preparations, variable anthocyanin doses, differences in food matrices and processing, and heterogeneous participant populations ranging from healthy individuals to overweight, prediabetic, and diabetic cohorts. These differences complicate comparisons across studies and may contribute to inconsistent metabolic responses. Furthermore, many trials assess acute postprandial responses or surrogate biomarkers rather than clinically meaningful long-term outcomes, such as sustained changes in HbA1c, diabetes progression, or cardiovascular events. In particular, acute postprandial effects observed predominantly in healthy adults should not be interpreted as evidence of sustained improvements in glycemic control or metabolic health in individuals with type 2 diabetes. Because metabolically healthy participants may differ from individuals with type 2 diabetes in glucose metabolism, insulin sensitivity, disease status, and concomitant medication use, findings from these acute studies cannot be directly extrapolated to clinical populations. The frequent use of mixed-berry or multi-component formulations further limits attribution of observed effects specifically to *R. nigrum*. Accordingly, the current evidence base should be regarded as supportive of biological plausibility and potential dietary benefit rather than sufficient evidence for therapeutic recommendations [86]. Fourth, potential interactions between blackcurrant polyphenols and antidiabetic or cardiovascular drugs, through glucose transport, endothelial function, platelet activity, and cytochrome-mediated metabolism, together with limited long-term safety data, warrant attention in polypharmacy [96]. Future work should prioritize standardized and chemically characterized blackcurrant formulations, with defined concentrations of major anthocyanins and other relevant polyphenols, together with standardized reporting of the cultivar, processing method, food matrix, and administered dose, and clinically relevant endpoints (HbA1c, insulin sensitivity, and cardiometabolic outcomes) in adequately powered, longer-term studies comparing blackcurrants to other anthocyanin sources. Such standardization is essential to improve comparability across studies, enable reproducible dose–response assessment, and facilitate translation of phytochemical composition into clinically meaningful exposure. Pharmacokinetic and metabolomic studies are needed to define which circulating metabolites mediate the metabolic effects, their tissue distribution, and their dose–response relationships, while precision-nutrition approaches integrating microbiome profiling may help explain and predict inter-individual variability in response.

## 7. Conclusions

*Ribes nigrum* shows promise as a multi-targeted dietary adjunct for glycemic control and metabolic health, acting through complementary mechanisms that converge several pathways involved in glucose and metabolic regulation, including AMPK-dependent insulin sensitization. However, the clinical evidence remains limited by small sample sizes, short intervention periods, heterogeneous formulations and anthocyanin doses, differences in food matrices and participant characteristics, and the frequent use of mixed-berry preparations. Moreover, most human studies assess acute postprandial responses or surrogate biomarkers rather than clinically meaningful long-term outcomes. Critically, the low bioavailability of parent anthocyanins and the uncertain contribution of microbiota-derived metabolites further complicate the translation of preclinical mechanisms into consistent clinical effects. Therefore, current evidence does not support specific therapeutic recommendations regarding dose, formulation, treatment duration, or modification of established antidiabetic therapy. *Ribes nigrum* should currently be considered a complementary dietary strategy rather than a replacement for evidence-based antidiabetic treatment. Realizing its potential will require standardized formulations, metabolite-level pharmacokinetic characterization, and adequately powered long-term randomized controlled trials that separate blackcurrant-specific effects from those of anthocyanins, in general, and evaluate clinically relevant endpoints.

## Figures and Tables

**Table 1:** Blackcurrant mechanisms of action.

Mechanism (Section)	Blackcurrant Action	Relevance to Diabetes/Metabolic Syndrome	Evidence Level and Model	Key Supporting Evidence
Carbohydrate digestion (4.1)	Moderate, competitive inhibition of α-amylase and stronger inhibition of α-glucosidase (potency comparable to acarbose; enhances acarbose)	Lower postprandial glucose	In vitro—purified enzymes/cell-free systems; human—acute randomized crossover trials	McDougall, 2005 [27]; Hui et al., 2020 [41]; Boath et al., 2012 [37]; Takikawa et al., 2010 [44]
Intestinal glucose transport (4.2)	Inhibition of intestinal glucose transport, principally GLUT2 (noncompetitive)	Slower glucose absorption	In vitro—heterologous transporter expression)	Kwon et al., 2007 [46]
AMPK/GLUT4 signalling (4.3)	↑ phospho-AMPKα; ↑ GLUT4 translocation in skeletal muscle	Improved peripheral glucose uptake and insulin sensitivity	Animal—diabetic mouse model	Iizuka et al., 2018 [47]
Incretin/GLP-1 (4.4)	↑ GLP-1 secretion (delphinidin-3-rutinoside as secretagogue); ↑ basal GLP-1 and PC1/3	Enhanced insulin secretion and satiety	Cell + animal	Iizuka et al., 2018 [47]; Kato et al., 2015 [48]; Tani et al., 2017 [49]
Oxidative stress/NF-κB (4.5)	Scavenges ROS; ↓ adipose inflammation, macrophage infiltration, and inflammatory cytokines (TNF-α, IL-1β)	Reduced inflammation-driven insulin resistance; β-cell protection	In vitro—purified enzymes/cell-free systems; Animal—diabetic/obesity-related mouse model; Human—observational/surrogate biomarker evidence	Benn et al., 2014 [50]; Shoelson, 2006 [51]
Microbiota–SCFA (4.6)	Alters microbiota composition; ↑ Lactobacilli and Bifidobacteria; SCFA-linked signalling	Improved glucose tolerance and gut barrier (indirect)	Animal—rodent model; Human—observational/surrogate biomarker evidence	Molan et al., 2014 [52]; Jaroslawska et al., 2016 [53]

**Table 2:** Human studies of blackcurrant or blackcurrant-containing interventions, classified by acute or long-term design.

#	Intervention	Study Type/Design	Sample	Main Outcomes	Reference/Registration/Limitations
1	Blackcurrant extract, 150/300/600 mg anthocyanins, before a meal	Acute. Randomized, double-blind crossover	*n* = 22 (14 M, 9 W); mean age 46 y	↓ postprandial glucose, insulin and incretin (GIP, GLP-1) at highest dose	Castro-Acosta et al., 2016 [86]; NCT01706653. Single-dose; acute endpoints only
2	Apple + blackcurrant polyphenol-rich drinks, before a meal	Acute. Randomized, double-blind crossover	Healthy adults	↓ postprandial glucose, insulin and GIP	Castro-Acosta et al., 2017 [87]. Combined polyphenol source; acute only
3	Blackcurrant purée ± fermented quinoa, 75 g	Acute. Randomized crossover	*n* = 26 (22 W, 4 M)	↓ postprandial glucose and insulin (0–60 min)	Lappi et al., 2021 [21]. Small, female-dominant; matrix variability
4	Blackcurrant + citrus (poly)phenols and fibre drink	Acute. Two randomized controlled trials	Healthy adults	Modest effects on glycaemia, gut hormones and appetite	Pinto et al., 2023 [26]. Combined source; acute only
5	New Zealand blackcurrant extract, 600 mg (acute) and 600 mg/day × 8 days	Acute and short-term. Double-blind, randomized, placebo-controlled	*n* = 12 (acute); *n* = 13 (8-day); overweight/obese	No acute effect on glucose/insulin; 8-day intake ↑ insulin sensitivity (+22%), ↓ CRP	Nolan et al., 2020 [89]. Small; distinguishes acute from short-term effects
6	Blackcurrant extract powder, 1500/672 mg/day	Long-term. Randomized controlled trial	*n* = 30; adults 20–60 y	↑ Lactobacilli and Bifidobacteria; ↓ β-glucuronidase, stool pH	Molan et al., 2014 [52]. Microbiota outcomes highly inter-individual
7	New Zealand blackcurrant extract, 3.2 mg/kg × 5 weeks	Long-term. Randomized placebo-controlled	*n* = 36; adults 20–60 y	↓ oxidative-stress markers; ↑ plasma FRAP	Hurst et al., 2020 [90]. Surrogate biomarkers only
8	Bilberry + blackcurrant anthocyanins, 320 mg × 12 weeks (mixed berry)	Long-term. Randomized controlled trial	*n* = 160; aged 40–75 y	↑ adipsin, ↓ visfatin; HbA1c change reported	L. Yang et al., 2021 [91]. Mixed-berry confounds attribution
9	Purified bilberry + blackcurrant anthocyanins, 320 mg/day × 12 weeks (mixed berry)	Long-term. Randomized, double-blind, placebo-controlled	*n* = 160; aged 40–75 y	↓ HbA1c, LDL-c, ApoB; improved insulin sensitivity	Yang et al., 2017 [92]. Mixed-berry limits specificity
10	Blackcurrant juice beverage, 369 mg anthocyanins × 7 days	Long-term (short). Double-blind, placebo-controlled	*n* = 24; adults 18–40 y	↑ plasma antioxidant capacity (ORAC)	Khan et al., 2014 [93]. Antioxidant marker only

## Data Availability

No new data were created or analyzed in this study. Data sharing is not applicable to this article.
